# Temporary Tracheal Compression by Dilated Functionally Normal Esophagus

**DOI:** 10.5811/cpcem.2018.3.37534

**Published:** 2018-04-18

**Authors:** Tatsuki Sengoku, Tomohiro Sonoo, Kei Kira, Yuji Takahashi, Hideki Hashimoto, Kensuke Nakamura

**Affiliations:** Hitachi General Hospital, Emergency Medicine and Critical Care Medicine Department, Jonancho, Hitachi-shi, Ibaraki, Japan

## CASE PRESENTATION

**Image 1:**
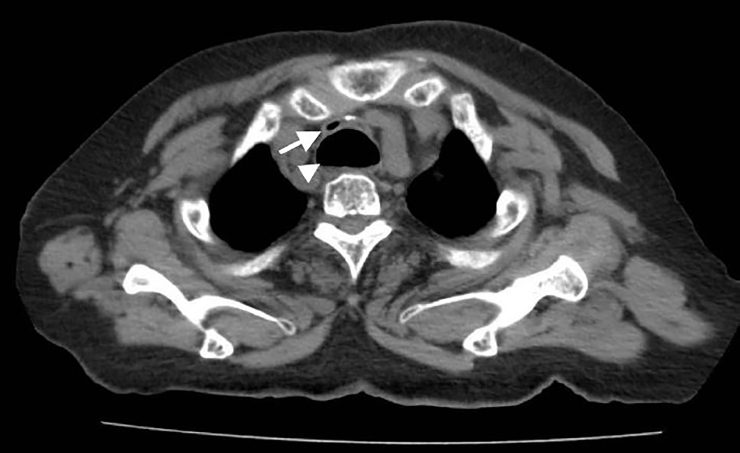
Compressed trachea (arrow) by dilated esophagus (arrowhead) at the level of the sternoclavicular joint.

**Image 2:**
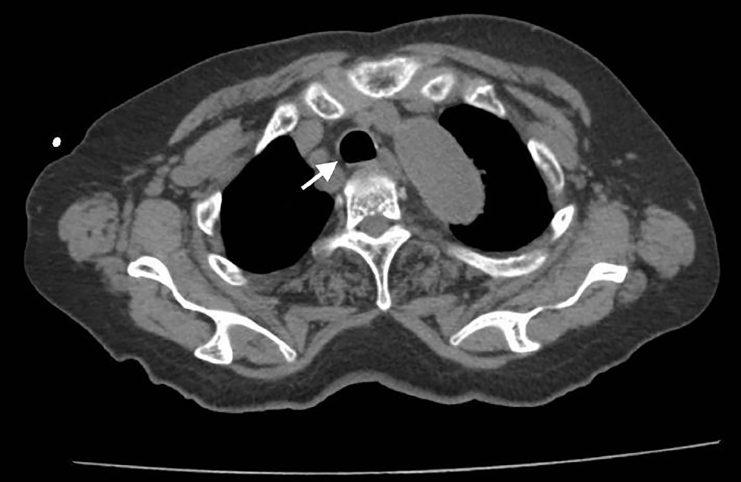
Repeated computed tomography with normal trachea (arrow).

**Image 3:**
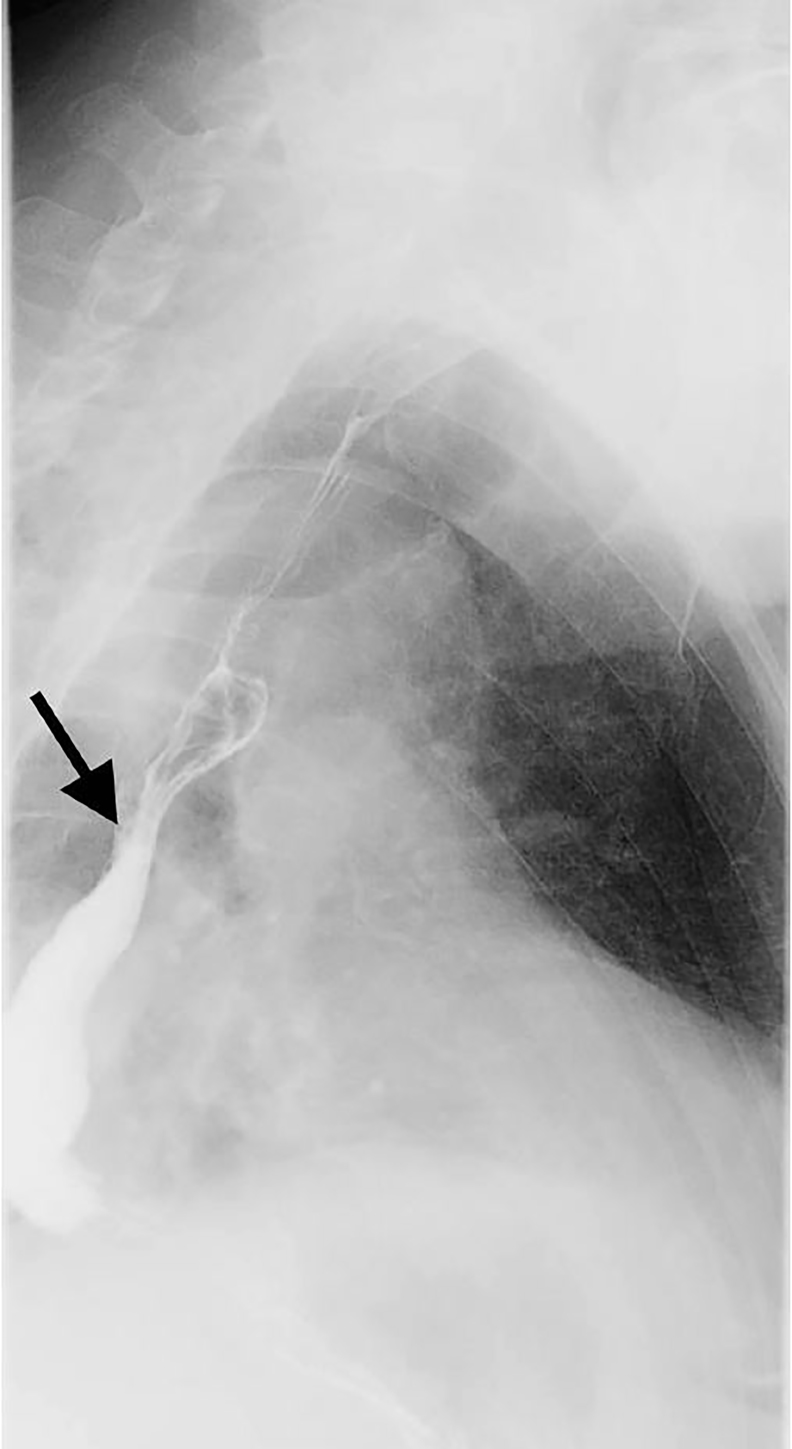
Esophagography revealed functionally normal esophagus (arrow).

An 80-year-old woman with a medical history of diabetes and duodenal cancer presented to our emergency department (ED) complaining of sudden severe dyspnea after vomiting. She was alert and oriented on arrival, but showed tachypnea and poor oxygenation. Inspiratory stridor was evident. A computed tomography (CT) revealed a dilated esophagus with food bolus and intraluminal air compressing the trachea at the level of the sternoclavicular joint (Image 1). Her symptoms improved after 30 minutes of rest and oxygen administration; however, she was admitted for observation. Repeat CT (Image 2) and esophagography (Image 3) were performed six days later and revealed no abnormality or evidence of esophageal dysfunction. The patient’s repeat CT, esophagography, and esophagogastroscopy revealed no abnormality or evidence of hiatal herniation or esophageal achalasia.

## DISCUSSION

This case presented reversible, severe tracheal compression by dilated esophagus with no functional abnormality. Cases of tracheal compression by dilated esophagus with structural diseases such as hiatal herniation^1^ or functional diseases such as esophageal achalasia^2^ have been reported. Dyspnea caused by esophageal achalasia is reportedly common in elderly women, who usually recover after treatment for esophageal achalasia.^2^ Unfortunately, functional evaluation of the esophagus is not performed in all cases. Some cases improve without treatment.^3^ Results obtained in this case suggest that a functionally normal esophagus can sometimes become sufficiently dilated to compress the upper airway, causing severe dyspnea. Spontaneous esophageal dilation is a rare cause of tracheal obstruction, but it is worth considering when no other cause is evident.

**CPC-EM Capsule**

What do we already know about this clinical entity?

Cases of tracheal compression by dilated esophagus with hiatal herniation or esophageal achalasia have been reported.

What is the major impact of the image(s)?

Reversible tracheal compression by dilated functionally normal esophagus was evident on the first CT.

How might this improve emergency medicine practice?

Spontaneous esophageal dilation is rare, but worth considering when we see the ED patients complaining of sudden severe dyspnea.

Documented patient informed consent and/or Institutional Review Board approval has been obtained and filed for publication of this case report.
